# Specific Bacterial Pathogen Phytosensing Is Enabled by a Synthetic Promoter-Transcription Factor System in Potato

**DOI:** 10.3389/fpls.2022.873480

**Published:** 2022-04-25

**Authors:** Ramona Persad-Russell, Mitra Mazarei, Tayler Marie Schimel, Lana Howe, Manuel J. Schmid, Tayebeh Kakeshpour, Caitlin N. Barnes, Holly Brabazon, Erin M. Seaberry, D. Nikki Reuter, Scott C. Lenaghan, C. Neal Stewart

**Affiliations:** ^1^Department of Plant Sciences, The University of Tennessee, Knoxville, Knoxville, TN, United States; ^2^Center for Agricultural Synthetic Biology, The University of Tennessee, Knoxville, Knoxville, TN, United States; ^3^Department of Food Science, The University of Tennessee, Knoxville, Knoxville, TN, United States

**Keywords:** phytosensors, pathogens, synthetic biology, synthetic promoter, reporter gene

## Abstract

Phytosensors are genetically engineered plant-based sensors that feature synthetic promoters fused to reporter genes to sense and report the presence of specific biotic and abiotic stressors on plants. However, when induced reporter gene output is below detectable limits, owing to relatively weak promoters, the phytosensor may not function as intended. Here, we show modifications to the system to amplify reporter gene signal by using a synthetic transcription factor gene driven by a plant pathogen-inducible synthetic promoter. The output signal was unambiguous green fluorescence when plants were infected by pathogenic bacteria. We produced and characterized a phytosensor with improved sensing to specific bacterial pathogens with targeted detection using spectral wavelengths specific to a fluorescence reporter at 3 m standoff detection. Previous attempts to create phytosensors revealed limitations in using innate plant promoters with low-inducible activity since they are not sufficient to produce a strong detectable fluorescence signal for standoff detection. To address this, we designed a pathogen-specific phytosensor using a synthetic promoter-transcription factor system: the S-Box *cis*-regulatory element which has low-inducible activity as a synthetic 4xS-Box promoter, and the Q-system transcription factor as an amplifier of reporter gene expression. This promoter-transcription factor system resulted in 6-fold amplification of the fluorescence after infection with a potato pathogen, which was detectable as early as 24 h post-bacterial infection. This novel bacterial pathogen-specific phytosensor potato plant demonstrates that the Q-system may be leveraged as a powerful orthogonal tool to amplify a relatively weak synthetic inducible promoter, enabling standoff detection of a previously undetectable fluorescence signal. Pathogen-specific phytosensors would be an important asset for real-time early detection of plant pathogens prior to the display of disease symptoms on crop plants.

## Introduction

Phytosensors are genetically engineered plants designed as real-time monitoring systems that can detect changes in environmental cues, environmental contaminants, or biological agents such as plant pathogens (Dangl and Jones, 2001; Kovalchuk and Kovalchuk, 2008; Liu and Stewart, 2016; Roper et al., 2021). The components for engineering synthetic promoters to develop phytosensors are products of innate biological systems such promoters or transcription factor genes that can sense stimuli or transduce signals (Liu and Stewart, 2016). To protect against pathogen infection, plants have evolved multiple lines of innate defense mechanisms. These defenses include basal resistance and resistance (R) gene-dependent resistance, both of which activate two types of systematic defense responses: induced systemic resistance (ISR) and systemic-acquired resistance (SAR) (Dangl and Jones, 2001; Vallad and Goodman, 2004). Key components of the plant defense system include highly conserved gene families that are controlled by signal transduction pathways, and *cis*-regulatory elements on the promoter regions of pathogen-inducible genes (Rushton et al., 2002; Liu et al., 2013b). These core regulatory elements can be utilized to design synthetic promoters as tools for developing phytosensors with improved sensitivity and specificity (Liu and Stewart, 2016). The basic foundation for constructing pathogen phytosensors includes an inducible promoter fused to a reporter gene that is activated when plants are infected by the pathogens. In our initial studies, the first pathogen-inducible reporting plants were constructed using native pathogen-inducible promoters (Kooshki et al., 2003). However, the reporter gene expression was too weak for spectral detection of fluorescence, restricting their potential use for standoff detection (Kooshki et al., 2003). In our subsequent studies, to increase the basal levels of reporter gene expression, synthetic promoters were designed using known core sequences of *cis*-regulatory promoter elements responsive to pathogen infection such as S-Box, W-Box, and GST1-Box (Rushton and Somssich, 1998; Mazarei et al., 2008); or elements responsive to the plant defense signal molecules salicylic acid, jasmonic acid, and ethylene (Liu et al., 2013a) were used to produce synthetic promoters supplemented with various combinations of *Cauliflower mosaic virus* (CaMV) 35S promoter enhancer domains fused to a reporter gene. The function of these phytosensors and their potential as real-time sensing reporters was demonstrated in stable transgenic *Arabidopsis* and tobacco plants (Mazarei et al., 2008; Liu et al., 2013a).

The S-Box *cis*-acting regulatory element plays a major role in elicitor responsiveness and mediates elicitor-enhanced expression of pathogen responsive genes (Kirsch et al., 2000). Although the S-box element is known to be induced in response to stimuli caused by fungal elicitor, *Phytophthora sojae* elicitor, oomycetes, and bacterial infection (Kirsch et al., 2000; Rushton et al., 2002), it conferred the lowest inducible expression when treated with phytohormones and chitin (Mazarei et al., 2008). This specificity of the S-Box *cis*-regulatory element in detecting pathogens and its relatively low-inducible activity limits its use as phytosensors. In order to improve its use as a pathogen-specific phytosensor with standoff detection capabilities, an effective reporter signal amplification system is required. In this study, we aimed to develop enhanced phytosensor capability using components from the quinic acid (*qa*) gene cluster of the fungus *Neurospora crassa*, known as the Q-system (Giles et al., 1985; Potter et al., 2010; Tang et al., 2011), to amplify the reporter gene signal and improve standoff detection of phytosensors. We previously showed that the Q-system transcriptional activator, QF, and its variant QF2, functions as an effective enhancer of reporter genes in transient expression experiments (Persad et al., 2020). We showed that this orthogonal tool for controlling gene expression constitutes a binary system, where the binding of QF or QF2 to a minimal promoter containing the QUAS sequence could activate and enhance gene expression up to 8-fold that was endowed by a synthetic promoter alone (Persad et al., 2020). Previous studies showed that the QF transcription factor had a lethal effect in transgenic *Drosophila* (Potter et al., 2010; Riabinina et al., 2015). On the other hand, the QF2 variant showed significant promise as an effective enhancer for generating stable transgenic plants (Persad et al., 2020). In this study, we further advanced this Q-system as an enhancer of reporter gene expression by developing stable transgenic potato (*Solanum tuberosum*) plants. By utilizing the Q-system QF2 variant driven by an S-Box-containing synthetic promoter along with the QUAS-binding domain, we demonstrated efficient bacterial pathogen phytosensing in this economically important crop. To our knowledge, this is the first report on the application of the Q-system as a signal amplification tool in stable transgenic plants.

## Materials and Methods

### Expression Vector Construction

**FIGURE 1:**
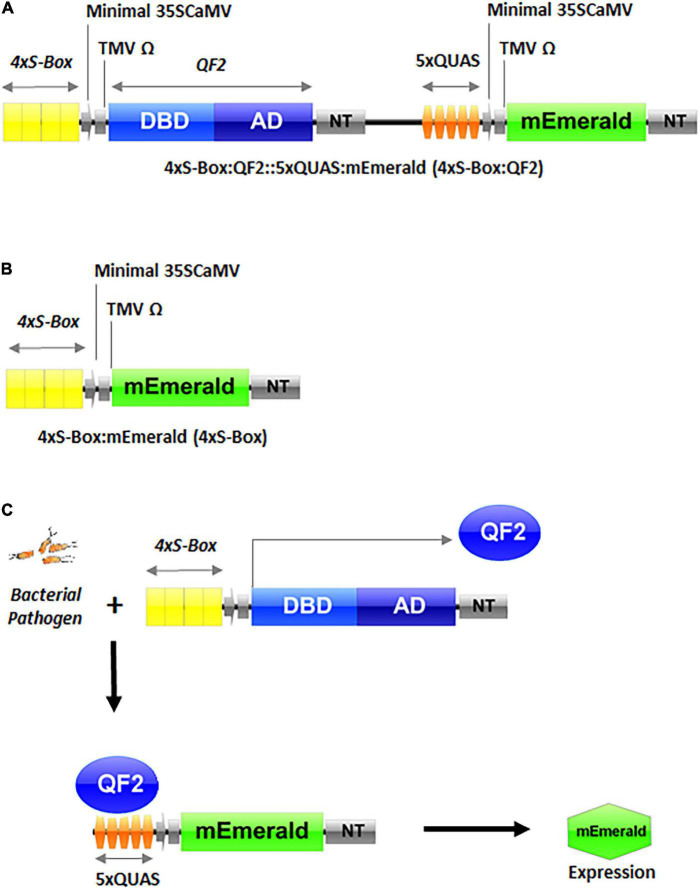
Schematic representation of inducible pathogen phytosensor constructs and illustration of the promoter-transcription factor functionality. **(A)** Phytosensor construct 4xS-Box:QF2::5xQUAS:mEmerald (4xS-Box:QF2) with the Q-system. **(B)** Pathogen phytosensor construct 4xS-Box:mEmerald (4xS-Box) without the Q-system. **(C)** Phytosensing mechanism illustrating the functional components of the Q-system. As shown earlier, the 4xS-Box synthetic promoter detects bacterial pathogen infection, resulting in the expression of QF2 protein. The subsequent binding of QF2 to the 5xQUAS promoter results in expression of the mEmerald reporter. DBD, DNA binding domain; AD, activation domain; QF2, Q-system transcription factor variant; QUAS, Q-system effector repeat promoter sequence; NT, nopaline synthase terminator; minimal 35SCaMV, minimal (−46) 35S promoter of the *Cauliflower mosaic virus* (CaMV); TMV Ω, 5′-leader sequence (Omega) of *Tobacco mosaic virus* (TMV); mEmerald, green fluorescent protein (GFP) reporter; S-Box, pathogen inducible *cis*-element.

Two expression vectors 4xS-Box:QF2::5xQUAS:mEmerald (with the Q-system amplifier QF2) and 4xS-Box:mEmerald (without the Q-system amplifier) were constructed and named: 4xS-Box:QF2 and 4xS-Box, respectively (Figures 1A,B). For vector construction, a 4x repeat of the S-Box sequence followed by the minimal *Cauliflower mosaic virus* (CaMV) 35S promoter and *Tobacco mosaic virus* (TMV) Ω (Omega: the 5′-leader sequence) regulatory sequences was first synthesized by Invitrogen GeneArt Gene Synthesis (Life Technologies, Regensburg, Germany), and then used to PCR amplify DNA fragments that were flanked by specific restriction sites, 5′*EcoRI-Mfe*I*-*4xSBox-minimal-35SCaMV-TMVΩ-*Avr*II*-Nco*I 3′ (Supplementary Table 1), for cloning into a destination vector. Primers used for cloning are provided in Supplementary Table 2. PCR-amplified DNA fragments were assembled using conventional restriction digest cloning into a destination vector. Restriction enzymes were supplied by New England Biolabs (NEB, Ipswich, MA, United States) and PCR fragments were amplified using Phusion high-fidelity DNA polymerase (NEB, #M0530S). For cloning, the binary expression vector pMTV-Nos: QF2 (Nos:QF2::5xQUAS:mEmerald), described in Persad et al. (2020), was used as a destination vector. The PCR and vector fragments were digested to produce compatible restriction sites for cloning and were gel purified using the Qiagen QIAquick gel extraction kit (Qiagen, Hilden, Germany, #28706X4), and ligated using T4 DNA ligase (NEB, #M0202S). The 4xS-Box:QF2 expression vector, was produced by digesting the fragments with restriction enzymes *Mfe*I and *Avr*II, to replace the Nos promoter on the destination vector with the 5′*MfeI-*4xS-Box-minimal35SCaMV-TMVΩ-*Avr*II 3′ PCR fragment. To produce the 4xS-Box expression vector, the destination vector was digested with restriction enzymes *Mfe*I and *Nco*I, to replace the Nos promoter and the Q-system components QF2 and 5xQUAS, with the 5′*MfeI-*4xS-Box-minimal35SCaMV-TMVΩ-*Nco*I 3′ PCR fragment.

### Generation of Transgenic Plants

Wild-type tissue culture potato plantlets (*Solanum tuberosum* cv. “Desiree”) were obtained from UW-Madison Wisconsin Seed Potato Certification Program (WSPCP) tissue culture laboratory and maintained in MS media (Plant Phytotec Labs, Lenexa, KS, United States, #M524). *Agrobacterium tumefaciens* strain LBA4404 carrying either the 4xS-Box:QF2 or 4xS-Box binary construct was used to infect internodal explants. Stable transgenic potato plants were produced following the *Agrobacterium*-mediated genetic transformation protocol (Chronis et al., 2014). Plantlets were selected on MS media containing antibiotic hygromycin and the presence of the transgene in the potato lines was confirmed by PCR analysis (Supplementary Figure 1). Three independent stably engineered potato plant lines, each containing 4xS-Box:QF2 (with the Q-system) or 4xS-Box (without the Q-system) were selected for bacterial pathogen infection. Primers used for the genotypic analysis of transgenic lines are provided in Supplementary Table 2.

### Bacterial Strains and Infection

*Streptomyces acidiscabie* (SA) was obtained from the American type culture collection (ATCC 49,004, U30), *Clavibacter michiganensis* subsp. *nebraskensis* (CMN) and *Pseudomonas marginalis* (PM) were obtained from Dr. Bonnie Ownley (University of Tennessee, Knoxville, TN, United States). For bacterial pathogen infection experiments, internodes from sterile control wild type and transgenic potato plants were grown in MS tissue culture media for 3 weeks and were transferred to potting mix for an additional 2–3 weeks under long-day growth chamber conditions [photoperiod of 16 h light (22°C) and 8 h darkness (16°C) regimen, at a photosynthetically active photon flux density (PPFD) of ∼ 200 μmol/m^2/^s^1^ and relative humidity of 70%]. Individual bacterial strains were grown at 28°C, shaking at 225 rpm overnight in BD*™* Bacto Tryptic Soy Broth medium (Soybean-Casein Digest Medium) (Fisher scientific, Waltham, MA, United States, # 2,11,822). Bacterial cells were centrifuged and resuspended in a 10 mM MgCl_2_ solution to an optical density OD_600_ of 0.3, corresponding to (3 × 10^8^ cfu/ml) for CMN, (3 × 10^8^ cfu/ml) for SA, and (1 × 10^8^ cfu/ml) for PM bacterial pathogens. Potato plant shoots were fully immersed into magenta boxes (PhytoTech Labs, Lenexa, KS, United States) containing bacterial solution and placed into a 20 l vacuum chamber (Best Value Vacs, Naperville, IL, United States). A vacuum pressure of approximately −84 kPa was applied for approximately 1 min four times, with regular agitation of the bacterial solution while plants were submerged. For mock control treatments, a 10 mM MgCl_2_ solution was used for the vacuum infiltration of plants. After infiltration, plants were well-watered, covered with a plastic dome and sealed with micropore tape to maintain high humidity, and returned to growth chamber conditions. In total of three independent bacterial infection experiments were performed. Each experiment contained three independent transgenic lines per construct and four biological replicate plants per construct.

### Heat Stress Treatment

Heat stress was performed in a controlled growth chamber environment. Plants were exposed to a transitory high-temperature regime over a five-day period (Supplementary Figure 4A). Heat stress was applied at a temperature of 44°C for 4 h. The temperature was allowed to gradually rise from normal growth conditions of 22°C to 44°C, over 30 min. After heat stress treatment the temperature was gradually returned to 22°C. The heat stress treatment was repeated daily for five days.

### Fluorescence Spectroscopy

Spectro-fluorescence measurements were obtained using Fluorolog^®^-3 spectrofluorometer, according to the manufacturer’s instructions (HORIBA Scientific, Austin, TX, version 3.8.0.60). An excitation (Ex) wavelength of 475 nm and emission (Em) range of 494–594 nm was used to obtain a fluorescence emission peak at 509 nm, specific to the mEmerald green fluorescent protein (GFP). Spectro-fluorescence signal intensity readings were collected from leaves of the same developmental stage for each biological replicate to ensure uniformity among the measured leaves in different plants. In total of three spectro-fluorescence readings were collected on each leaf as technical replicates, in order to account for the positional error.

### RNA Extraction, cDNA Synthesis, and Quantitative Reverse Transcriptase-PCR

Plant tissue samples were pooled from each spectro-fluorescence analyzed spot on a single leaf for RNA extraction for each biological replicate. RNA extraction, cDNA synthesis, and subsequent quantitative reverse transcriptase-PCR (qRT-PCR) was performed as outlined in Persad et al. (2020). qRT-PCR was performed for three biological replicates (*n* = 3) per treatment. For each biological replicate qRT-PCR was performed with three technical replicates. Transcript levels were normalized to potato elongation factor 1-alpha gene. Primers used for qRT-PCR are provided in Supplementary Table 2.

### Confocal Microscopy

Potato leaf sections were observed using an Olympus Fluoview1200 confocal microscope (Olympus, Center Valley, PA, United States) to visualize mEmerald GFP reporter expression, using an Ex/Em wavelength of 487/509 nm. Chlorophyll autofluorescence was excited at 543 nm and detected at 667 nm. For comparison, images were acquired using the same laser parameters.

### Fluorescence-Induced Laser Projector

Fluorescence-induced laser projector (FILP) imaging of plants expressing mEmerald GFP were acquired using the 465 nm excitation laser and 525/50 nm emission filter with 200 ms exposure at 0.80 watts laser power. Chlorophyll images were acquired using the 465 nm excitation laser and the 680/50 emission filter. Imaging parameters were determined as described previously (Rigoulot et al., 2021). Images were processed and data were extracted using image processing and analysis in Java (ImageJ, version 1.52a) (Schneider et al., 2012). For comparison, images were acquired using the same laser parameters, and images were processed using the same ImageJ settings.

### Statistical Analysis

Statistical analysis was performed using IBM SPSS software (IBM Corp. Released 2017. Version 25.0). Differences were considered to be statistically significant using a confidence level of *p* < 0.05. Analysis of full spectral data was performed using a one-way repeated measures ANOVA, which considers all data points along the spectrum when determining statistical significance. For analysis of fluorescence spectral data at 509 nm, a one-way ANOVA was performed, followed by *post hoc* analysis using Tukey HSD. For qRT-PCR, a one-way ANOVA was performed with the least significant difference (LSD) *post hoc* analysis.

## Results

### Q-System as a Signal Amplifier for Inducible Pathogen Promoters

In total three independent stably transgenic potato plant lines were produced for each construct: 4xS-Box:QF2 (with the Q-system) or 4xS-Box (without the Q-system) (Supplementary Figure 1). These lines were morphologically indistinguishable from their non-transgenic parent lines (“Desiree”). Among the three pathogens *Streptomyces acidiscabie* (SA), *Clavibacter michiganensis* subsp. *nebraskensis* (CMN), and *Pseudomonas marginalis* (PM) that were used to treat leaves (Supplementary Figure 2A), SA, or CMN led to a normal-sensitive symptom of “leaf spots” associated with initial chlorosis followed by water-soaked and necrotic symptoms of the tissue (susceptible reaction) (Supplementary Figure 2B). No symptom development was evident following inoculation with bacterial pathogen PM (Supplementary Figure 2B).

**FIGURE 2:**
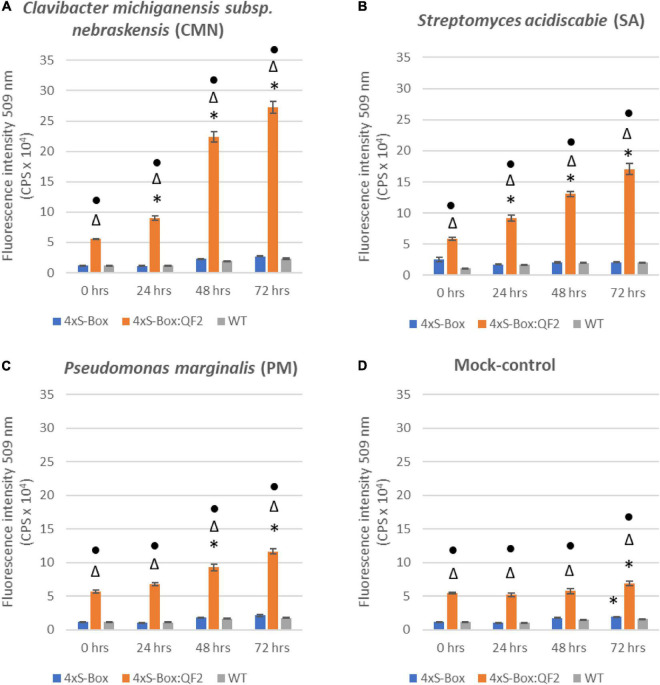
Time course spectro-fluorescence analysis of enhanced pathogen phytosensor. Spectral data represent mEmerald emission measured at 509 nm emission obtained with a 475 nm excitation wavelength. mEmerald fluorescence intensity measured for transgenic phytosensor 4xS-Box:QF2 (with Q-system) and 4xS-Box (without Q-system), and WT (wild-type) plants at 0, 24, 48, and 72 h post inoculation with bacterial pathogens: **(A)** *Clavibacter michiganensis* subsp. *nebraskensis* (CMN), **(B)** *Streptomyces acidiscabie* (SA), **(C)** *Pseudomonas marginalis* (PM), and **(D)** 10 mM MgCl_2_ (mock-control). Plants were infiltrated with a bacterial inoculum of OD_600_ = 0.3, corresponding to colony forming units (cfu) of 3 × 10^8^ cfu/ml CMN, 3 × 10^8^ cfu/ml SA, and 1 × 10^8^ cfu/ml PM. Statistically significant differences determined using ANOVA, Tukey HSD *post hoc* analysis. Significant differences indicated by: * from 0 h (before treatment); △ from WT; • from 4xS-Box (*p* < 0.05). Three independent experiments with three biological replicate plants (*n* = 3) per experiment was performed. In total three technical replicate spectral readings were collected on a single leaf for each biological replicate per experiment to account for positional error. Data represent mean ± standard error of 27 spectral readings.

**FIGURE 3:**
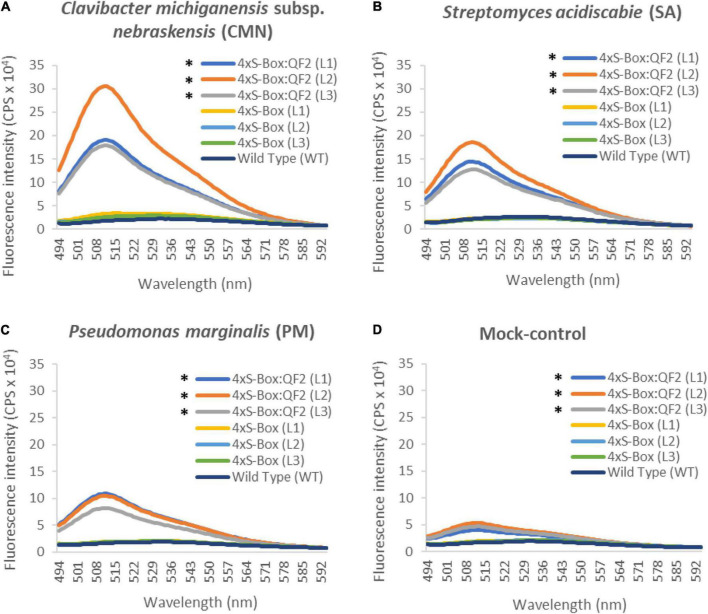
Spectro-fluorescence signal amplification in pathogen phytosensors. Full spectral analysis from transgenic phytosensor 4xS-Box:QF2 (with Q-system) and 4xS-Box (without Q-system), and wild-type pants inoculated with bacterial pathogens **(A)** *Clavibacter michiganensis* subsp. *nebraskensis* (CMN), **(B)** *Streptomyces acidiscabie* (SA), **(C)** *Pseudomonas marginalis* (PM), and **(D)** 10 mM MgCl_2_ (mock-control). Plants were infiltrated with a bacterial inoculum of OD_600_ = 0.3, corresponding to colony forming units (cfu) of 3 × 10^8^ cfu/ml CMN, 3 × 10^8^ cfu/ml SA, and 1 × 10^8^ cfu/ml PM. Spectral data represent mEmerald emission of treated leaves at 72 h post inoculation. Spectral data measured at 475 nm excitation and 494 − 594 nm emission wavelengths. Statistical significance determined for all data points across spectrum using repeated measures ANOVA, Tukey HSD *post hoc* analysis. Asterisks (*) indicate significant difference (*p* < 0.05), compared with WT (wild type) and 4xS-Box. In total three independent experiments with four biological replicate plants (*n* = 4) per experiment was performed. In total three independent lines (L1, L2, and L3) were tested for each construct. In total three technical replicate spectral readings were collected on a single leaf for each biological replicate per experiment to account for positional error. Data represent mean ± standard error of 36 spectral readings.

**FIGURE 4:**
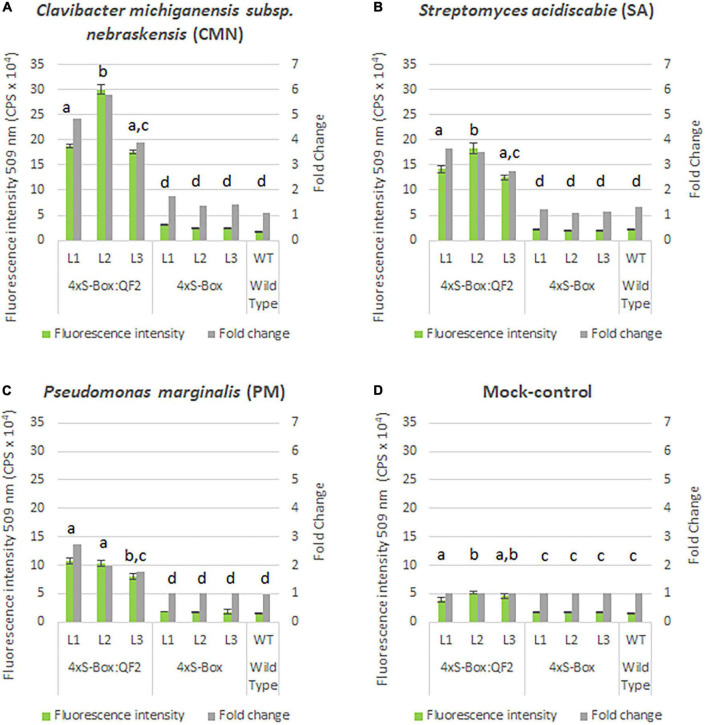
Spectral analysis of mEmerald emission in pathogen phytosensors. Spectral data represent mEmerald peak emission for transgenic phytosensor 4xS-Box:QF2 (with Q-system) and 4xS-Box (without Q-system), and WT (wild type) plants at 72 h post inoculation with bacterial pathogens: **(A)** *Clavibacter michiganensis* subsp. *nebraskensis* (CMN), **(B)** *Streptomyces acidiscabie* (SA), **(C)** *Pseudomonas marginalis* (PM), and **(D)** 10 mM MgCl_2_ (mock-control). Plants were infiltrated with a bacterial inoculum of OD_600_ = 0.3, corresponding to colony forming units (cfu) of 3 × 10^8^ cfu/ml CMN, 3 × 10^8^ cfu/ml SA, and 1 × 10^8^ cfu/ml PM. Spectral data measured at 509 nm emission obtained with a 475 nm excitation wavelength. Statistically significant differences determined using ANOVA, Tukey HSD *post hoc* analysis: groups with different letters show a significant difference (*p* < 0.05). Three independent experiments with four biological replicate plants (*n* = 4) per experiment was performed. In total three independent lines (L1, L2, and L3) were tested for each construct. In total three technical replicate spectral readings were collected on a single leaf for each biological replicate per experiment to account for positional error. Data represent mean ± standard error of 36 spectral readings. Fold change relative to mock-control treatment.

Observations from initial time-course pathogen inoculation experiments showed a steady increase in mEmerald emission from 24, 48, and 72 hpi (Figure 2 and Supplementary Figure 3). Quantitative analysis of full spectral data indicated that all three independent transgenic 4xS-Box:QF2 lines (L1, L2, L3) had a statistically significant (*p* < 0.05) increase in mEmerald emissions when compared with the 4xS-Box lines (L1, L2, L3), after inoculation with bacterial pathogens CMN, SA, and PM (Figure 3). There was no statistically significant difference in the reporter signal for 4xS-Box plants when compared with the background signal readings obtained for wild-type plants, under all treatments (Figure 3). Furthermore, analysis of spectro-fluorescence signal intensity data at 475 nm excitation and 509 nm emission wavelength (specific emission peak for mEmerald GFP), showed a statistically significant (*p* < 0.05) fold increase in reporter emission after pathogen inoculation for the 4xS-Box:QF2 lines when compared to 4xS-Box lines, under all treatments (Figure 4). The 4xS-Box:QF2 lines treated with CMN pathogen showed an average of ∼ 5-fold amplification of reporter signal (L1, 3.9; L2, 5.8; L3, 4.8) (Figure 4A). Similarly, 4xS-Box:QF2 lines treated with SA pathogen, showed an average of ∼ 3-fold amplification of reporter signal (L1, 3.7; L2, 3.5; L3, 2.7) (Figure 4B). A similar trend was also observed for the 4xS-Box:QF2 lines treated with PM pathogen with an average of ∼ 2-fold amplification of reporter signal (L1, 2.8; L2, 2.0; L3, 1.8) (Figure 4C). There was no statistically significant difference in the reporter signal for 4xS-Box plants when compared with background fluorescence obtained for wild-type plants, under all treatments (Figure 4).

**FIGURE 5:**
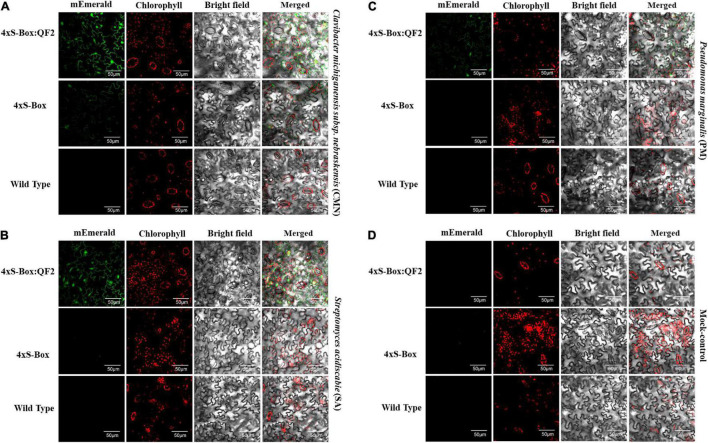
Confocal micrographs of the pathogen phytosensors. Confocal micrographs showing expression of mEmerald fluorescent protein in leaf cells of transgenic phytosensor 4xS-Box:QF2 (with Q-system), 4xS-Box (without Q-system), and wild type plants inoculated with bacterial pathogens **(A)** *Clavibacter michiganensis* subsp. *nebraskensis* (CMN), **(B)** *Streptomyces acidiscabie* (SA), **(C)** *Pseudomonas marginalis* (PM), and **(D)** 10 mM MgCl_2_ (mock-control). Plants were infiltrated with a bacterial inoculum of OD_600_ = 0.3, corresponding to colony forming units (cfu) of 3 × 10^8^ cfu/ml CMN, 3 × 10^8^ cfu/ml SA, and 1 × 10^8^ cfu/ml PM. Images were recorded at 72 h post inoculation. In total three independent experiments with four biological replicate plants (*n* = 4) per experiment was performed. Images representative of one independent line per construct. The intensity of mEmerald was increased in the cells of phytosensors with the Q-system. Scale bar: 50 μm, magnification: 60×.

**FIGURE 6:**
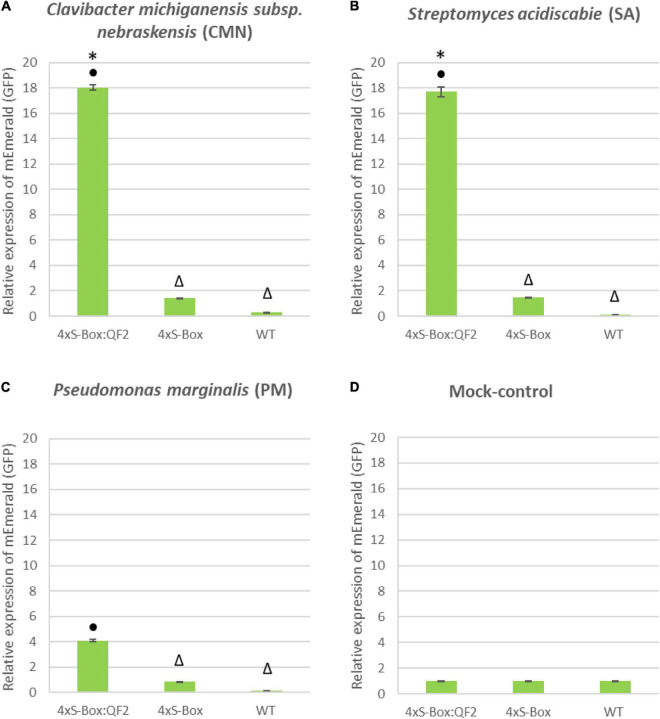
qRT-PCR expression analysis of mEmerald in 4xS-Box and 4xS-Box:QF2 treated with bacterial pathogens. RNA extracted from leaves of 6-week-old stable transgenic 4xS-Box and 4xS-Box:QF2 potato plant 72 h post inoculation with bacterial pathogens: **(A)** *Clavibacter michiganensis subsp. nebraskensis* (CMN), **(B)** *Streptomyces acidiscabie* (SA), and **(C)** *Pseudomonas marginalis* (PM). **(D)** Mock control, plants treated with 10 mM MgCl_2_ solution. Statistically significant differences determined using ANOVA, LSD *post hoc* analysis. Statistically significant (*p* < 0.05) differences in mEmerald expression between lines indicated by: • from WT (wild type); * from 4xS-Box and △ from 4xS-Box:QF2. Transcript levels were normalized to potato elongation factor 1-alpha gene. RNA extracted from three biological replicates (*n* = 3), with three technical replicated pooled from each leaf. Three technical replicate qRT-PCR reaction was performed per sample. Data represent mean ± standard error of three replicates.

Overall, the relative enhancement of reporter signal from the 4xS-Box:QF2 phytosensors was at least ∼ 2 times greater than the 4xS-Box phytosensors, depending on the type of bacterial pathogens (Figure 4). The effect of the QF2 activator at enhancing reporter expression was more dramatic for the 4xS-Box:QF2 line L2 (∼ 6-fold increase) treated with the CMN pathogen (Figures 3, 4). Furthermore, qualitative microscopy analysis confirmed a strong mEmerald expression for the 4xS-Box:QF2 phytosensor compared to the 4xS-Box phytosensor for the pathogen treatments (Figure 5). qRT-PCR expression analysis of mEmerald in 4xS-Box and 4xS-Box:QF2 lines treated with various bacterial pathogens show the same trend in reporter expression for CMN and SA pathogens (Figure 6). 4xS-Box:QF2 phytosensor plants had a statistically significant (*p* < 0.05) fold increase mEmreald gene expression after treatment with CMN (12.5-fold) and SA (12-fold) pathogens, when compared to 4xS-Box treated plants (Figures 6A,B). No significant increase in reporter expression was observed for 4xS-Box under all pathogen treatments (Figure 6).

### Fluorescence Imaging of Phytosensors

**FIGURE 7:**
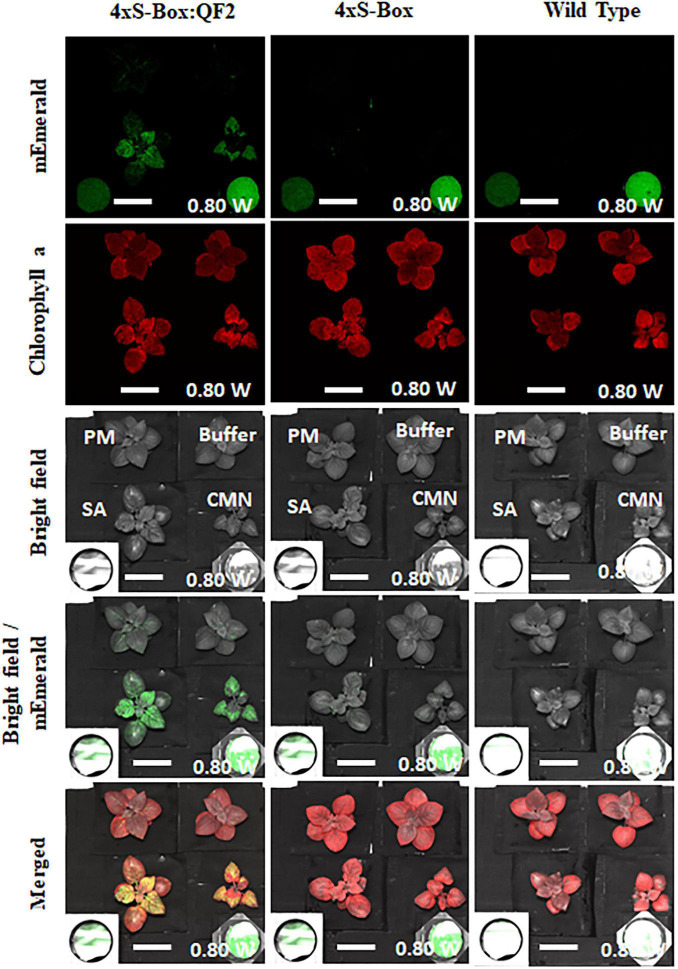
Detection of fluorescence in phytosensors for standoff detection application. Images acquired using the fluorescence-induced laser projector (FILP) system. FILP images showing detection of fluorescence in pathogen phytosensors with the Q-system. In total of 4xS-Box:QF2 (with Q-system), 4xS-Box (without Q-system), and wild-type plants were inoculated with bacterial pathogens *Clavibacter michiganensis* subsp. *nebraskensis* (CMN), *Streptomyces acidiscabie* (SA), *Pseudomonas marginalis* (PM), and 10 mM MgCl_2_ (mock-control). Plants were infiltrated with a bacterial inoculum of OD_600_ = 0.3, corresponding to colony forming units (cfu) of 3 × 10^8^ cfu/ml CMN, 3 × 10^8^ cfu/ml SA, and 1 × 10^8^ cfu/ml PM. Three independent experiments with four biological replicate plants (*n* = 4) per experiment was performed. Images representative of one independent line per construct. Images show the ability to detect mEmerald at 72 h after bacterial inoculation. Pixel intensity at 0 h (before treatment) was subtracted to normalize against background emission before treatment. Images processed with imageJ using the same brightness and contrast settings. The fluorescent images were acquired at a distance of 3 m from laser source with 200 ms exposure time at 0.80 watts laser power. mEmerald GFP images were acquired using the 465 nm excitation laser and the 525/50 nm emission filter. Chlorophyll images were acquired using the 465 nm excitation laser and the 680/50 emission filter. Scale bar: 5 cm.

Initial standoff detection experiments at a distance of 3 m showed an increase in mEmerald emission at 24, 48, and 72 hpi with pathogens CMN, SA, and PM (Supplementary Figure 3). The observed increase in reporter emission was more pronounced for 4xS-Box:QF2 plants inoculated with pathogens, compared with the 4 × S-Box plants (Figure 7).

Qualitative FILP observations of the sensitivity of the 4xS-Box:QF2 phytosensors to varying bacterial inoculum concentrations, showed a visible reporter signal for concentrations ranging from 6 × 10^6^ to 2 × 10^5^ cfu/ml, with a visibly weaker signal observed for lower concentrations (<8 × 10^5^ cfu/ml) at 72 hpi (Supplementary Figure 4). Lower dilutions (<8 × 10^5^ cfu/ml) resulted in weaker reporter signals, yet visible at 72 hpi (Supplementary Figure 4). Furthermore, after exposure to transitory heat stress conditions (Supplementary Figure 5A), quantitative analysis by FILP-imaging system (Supplementary Figure 5B) and spectro-fluorescence (Supplementary Figure 5C) showed that the 4xS-Box:QF2 plants had no significant increase in reporter amplification.

## Discussion

Early detection of pathogen infection before crop damage is an ongoing challenge within the agricultural sector (Savary et al., 2012). Phytosensors can provide early detection of diseases, however, their potentially weak production of unique spectra may restrict their practical use for standoff detection. Therefore, we designed a paired synthetic promoter and synthetic transcriptional activator to attain a robust reporter signal that was conditional upon pathogen infection. The resulting potato plant represents the first phytosensor device using such a design. Our study is the first to examine the QF2 Q-system variant to amplify the reporter signal of a weak inducible S-Box synthetic promoter in stably engineered plants. Since the S-Box *cis*-regulatory sequence is known to induce in response to stimuli caused by a bacterial infection (Kirsch et al., 2000; Rushton et al., 2002), we investigated reporter amplification in 4xS-Box:QF2 (with the Q-system) and 4xS-Box (without the Q-system) phytosensors in response to bacterial pathogens: CMN, SA, and PM.

Notably, the Q-system enhanced 4xS-Box:QF2 phytosensors provided a significantly higher reporter signal after treatment with the bacterial pathogens when compared with the 4xS-Box phytosensor without the Q-system enhancer (Figures 2–4). For the 4xS-Box:QF2 phytosensors, the inducible reporter expression was highest for the CMN, followed by the SA, which is suggestive of their known pathogenicity as leaf spot disease pathogens (Lambert and Loria, 1989; Eichenlaub and Gartemann, 2011; Charkowski et al., 2020; Ismail et al., 2020). The relatively lower reporter increase observed for PM pathogen was predicted, since the PM pathogen is known for causing soft rot disease on potato tubers and is not considered as leaf spot causing disease (Li et al., 2007). The relative increase in mEmerald reporter signal for the 4xS-Box:QF2 in comparison to the 4xS-Box phytosensor, confirmed at the cellular level (Figure 5), provides further evidence of the effectiveness of the Q-system in amplifying the reporter signal from weak inducible promoters.

The synthetic inducible promoter used for this study was designed as a homotetrameric repeat of the S-Box *cis*-regulatory sequence arranged in a head-to-tail orientation, followed by the minimal CaMV 35S promoter and a downstream TMV sequence (Figure 1). Elements from the CaMV 35S promoter and TMV promoter are well-known enhancers of transcription (Kay et al., 1987; Fang et al., 1989; Gallie, 2002). For the CMN-treated 4xS-Box phytosensors without the Q-system enhancer, although low amount of mEmerald protein were visible in leaf tissue under confocal microscopy (Figure 5), the weak signal detected with fluorescence spectroscopy was not significantly different from the background fluorescence obtained for wild-type plants that were treated with the same pathogen (Figures 2–4). This corresponds to observations made in our earlier studies in which phytosensors developed with either the native pathogen-inducible promoter sequence, or with a synthetic tetramer promoter supplemented with CaMV 35S enhancer elements, showed low-inducible reporter expression that could be detected with western blot and fluorometric analysis, but the signal was too weak for spectral detection (Kooshki et al., 2003; Mazarei et al., 2008). Here, we demonstrated that the 4xS-Box synthetic promoter supplemented with the minimal CaMV 35S promoter and an additional downstream TMV-enhancer sequence was still not sufficient to provide an enhanced reporter signal that could be detected by the fluorescence spectroscopy.

Our Q-system-enhanced pathogen phytosensor, 4xS-Box:QF2 was designed to activate its function when the 4xS-Box synthetic promoter detects bacterial pathogen infection, resulting in the expression of QF2 protein which then binds to the 5xQUAS recognition sequence that drives the expression of the mEmerald reporter (Figure 1C). The 5xQUAS synthetic promoter, designed from the innate QUAS *Neurospora crassa* sequence, allows specific binding of QF2 transcriptional activator proteins (Riabinina et al., 2015; Persad et al., 2020). Standoff detection capabilities of our 4xS-Box:QF2 pathogen phytosensor, indicated that an mEmerald fluorescence signal was detected in potato leaves as early as 24 hpi (Figure 2 and Supplementary Figure 3), well before plants are expected to show post-symptomatic responses.

Generally, plant disease remote sensing utilizes hyperspectral imaging that depends on electromagnetic radiation and changes in the amount of light that is absorbed and reflected by leaf pigments such as chlorophyll and xanthophyll (Nilsson, 1995; Blackburn, 2006; Mahlein et al., 2012; Lowe et al., 2017). Other remote-sensing techniques include thermography and chlorophyll fluorescence sensors (Mahlein et al., 2012). However, for successful detection and identification of plant diseases, these techniques require the development of spectral indices that are often difficult to determine and standardize owing to heterogenous environmental conditions and variation in the plant morphology (Blackburn, 2006; Mahlein et al., 2013). The advantage of using synthetic inducible phytosensors with enhanced fluorescence reporter expression is that a real-time early detection of plant diseases can be remotely sensed using spectral wavelengths that are specific to the transgenic reporter gene. Enhancing the fluorescence reporter expression makes it easier to distinguish an activated phytosensor among the background fluorescence emitted from non-transgenic (non-phytosensing) plants. In addition, the sensitivity of the Q-system enhanced 4xS-Box:QF2 phytosensor (Supplementary Figure 4) and its specificity to biotic stress as observed after exposure to abiotic heat stress (Supplementary Figure 5), significantly improves its potential use as a sensing and reporting platform for detecting plant diseases. Future research of the Q-system-enhanced phytosensor in the field would provide further insight on its practicality and use. Ultimately, the end goal would be to employ the use of pathogen phytosensors for monitoring plant diseases affecting field crops in an agricultural setting. Since the occurrence of plant diseases often require a combination of specific environmental factors, crops grown in the field often exhibit a patchy distribution of diseases (Colhoun, 1973; Mahlein et al., 2012). Remote-sensing applications such as light detection and ranging (LiDAR), fluorescence spectroscopy, thermal spectroscopy, and hyperspectral imaging have long been implemented for improving precision agriculture (Gebbers and Adamchuk, 2010; Mulla, 2013). With the development of our specific bacterial pathogen phytosensor, we can further improve precision agriculture and integrate pest management strategies with the site-specific application of pesticides.

## Conclusion

We have demonstrated the application of the Q-system transcriptional activator QF2 variant in stable transgenic plants for translational research. We showed that the Q-system QF2 variant could be effectively used for the development of enhanced pathogen phytosensing potato plants, enabling standoff detection at a distance of three meters using laser-imaging technology. In this new era of agricultural synthetic biology, our study presents an improved phytosensing and reporting platform, with potential application for developing phytosensors specific to pathogens and other targets such as explosives, radiation, heavy metals, and other environmental toxins, and contaminants.

## Data Availability Statement

The datasets presented in this study can be found in online repositories. The names of the repository/repositories and accession number(s) can be found in the article/Supplementary Material.

## Author Contributions

RP-R analyzed data, produced figures, wrote the manuscript, cloned genetic constructs, designed, led, and performed experiments and microscopy. MM participated in the experimental design and bacterial pathogenicity disease interpretations. RP-R and DR extracted DNA and performed colony PCR. MS, CB, HB, and ES produced transgenic plants. RP-R, LH, and MS maintained tissue culture and plants. LH, MS, TK, and MM assisted with the pathogen inoculation experiments and collecting spectral data. LH assisted with heat stress and pathogen sensitivity experiments. TS, LH, and MS assisted with FILP imaging. RP-R and TS extracted data from FILP images. CS and SL conceived the study and acquired funding. CS, SL, and MM assisted with the revisions and edits to the manuscript. All authors read and have agreed to the published version of the manuscript.

## Author Disclaimer

The views, opinions, and/or findings expressed are those of the authors and should not be interpreted as representing the official views or policies of the Department of Defense or the United States Government (Approved for Public Release, Distribution Unlimited).

## Conflict of Interest

The authors declare that the research was conducted in the absence of any commercial or financial relationships that could be construed as a potential conflict of interest.

## Publisher’s Note

All claims expressed in this article are solely those of the authors and do not necessarily represent those of their affiliated organizations, or those of the publisher, the editors and the reviewers. Any product that may be evaluated in this article, or claim that may be made by its manufacturer, is not guaranteed or endorsed by the publisher.
