# Supplementary material for: Specific Bacterial Pathogen Phytosensing Is Enabled by a Synthetic Promoter-Transcription Factor System in Potato
Source: Front Plant Sci. 2022 Apr 25;13:873480. doi: 10.3389/fpls.2022.873480 (PMC9083229; doi:10.3389/fpls.2022.873480)
Supplement: Supplementary file 1 [file Data_Sheet_1.docx]

**Supplementary data**

**Specific bacterial pathogen phytosensing is enabled by a synthetic promoter-transcription factor system in potato**

Ramona Persad-Russell*^1,2^*, Mitra Mazarei*^1,2^*, Tayler Marie Schimel*^2,3^*, Lana Howe*^1,2^*, Manuel J. Schmid*^1,2^*, Tayebeh Kakeshpour*^1,2^*, Caitlin N. Barnes*^1,2^*, Holly Brabazon*^1,2^*, Erin M. Seaberry*^1,2^*, D. Nikki Reuter*^2,3^*, Scott C. Lenaghan*^2,3*^* and C. Neal Stewart, Jr*^1,2*^*

*^1^ Department of Plant Sciences, University of Tennessee, Knoxville, Tennessee, USA*

*^2^Center for Agricultural Synthetic Biology, University of Tennessee, Knoxville, Tennessee, USA*

*^3^ Department of Food Science, University of Tennessee, Knoxville, Tennessee, USA*

**^*^Correspondence:** C. Neal Stewart, Jr. and Scott C. Lenaghan

Tel: 865-974-6487; Emails: nealstewart@utk.edu (CNS), slenagha@utk.edu (SCL)


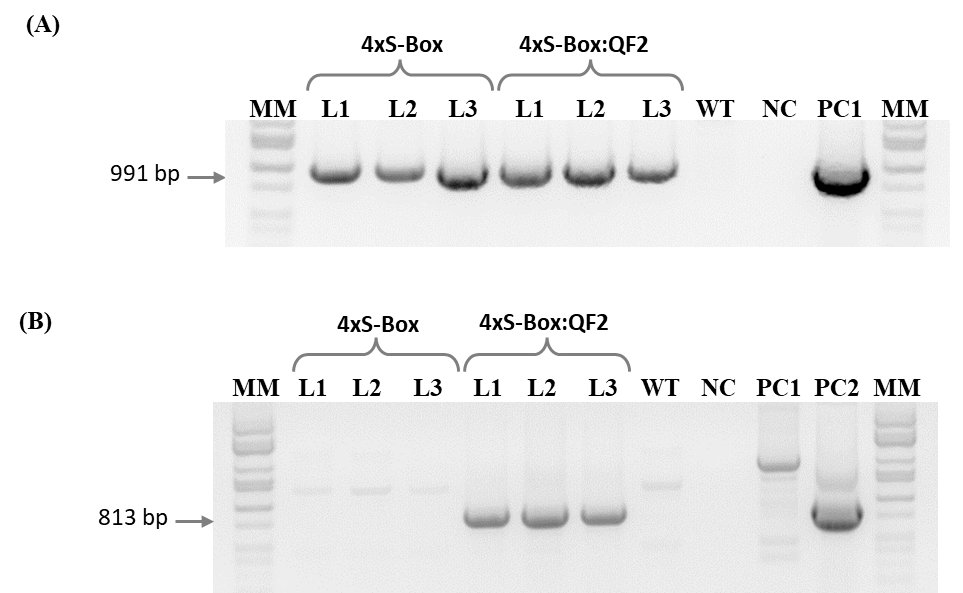


**Figure S1: Transgene insertion confirmation for 4xS-Box and 4xS-Box:QF2 lines.**

PCR analysis of genomic DNA extracted from leaves of 6-week-old stable transgenic 4xS-Box and 4xS-Box:QF2 potato plant lines (L1, L2 and L3 indicated). WT, wild type; MM, molecular marker; NC, negative control (no DNA template); PC1, positive control template 4xS-Box plasmid DNA; PC2, positive control template 4xS-Box:QF2 plasmid DNA. Expected amplified DNA band size indicate presence of **(A)** mEmerald gene (991 bp); and the presence or absence of Q-system transcription factor **(B)** where the QF2 transcription factor (813 bp) is present in 4xS-Box:QF2 plant lines but not in 4xS-Box plant line.


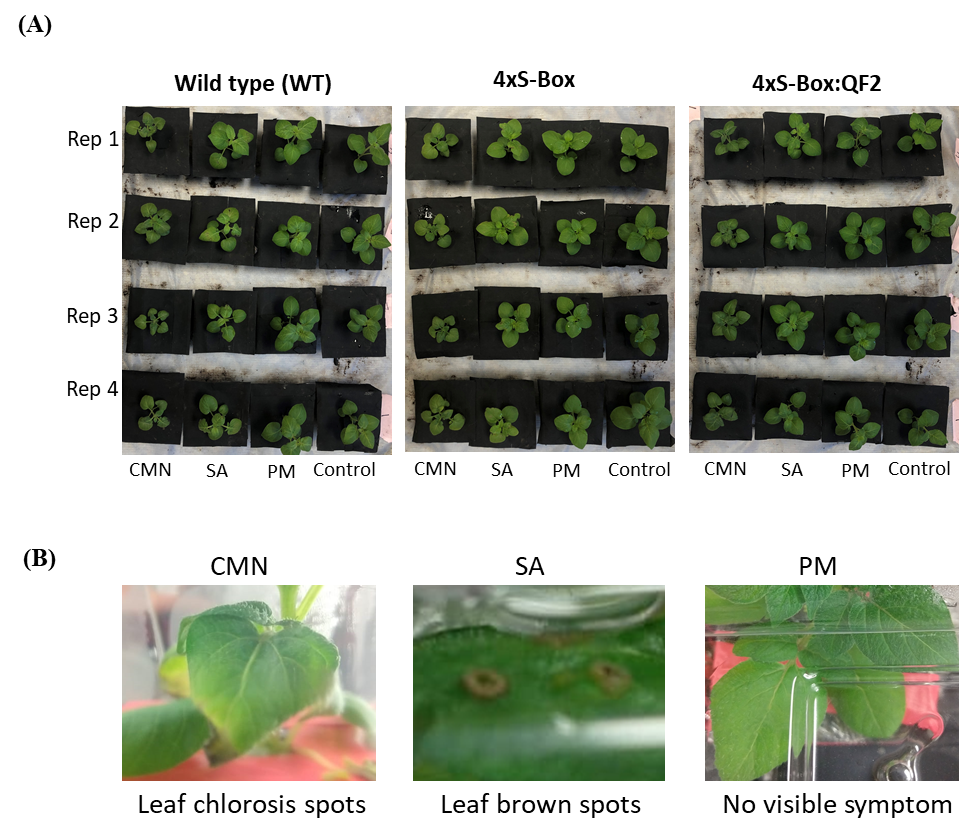


**Figure S2: Bacterial pathogenicity tests.**

Plants were treated by vacuum infiltrating bacterial pathogens bacterial pathogens resuspended at OD_600_ = 0.30: *Clavibacter michiganensis* subsp. *nebraskensis* (CMN), *Streptomyces acidiscabie* (SA), and *Pseudomonas marginalis* (PM). **(A)** Images shown represent symptoms observed 72 h after inoculation. One line represented for each genotype 4xS-Box and 4xS-Box:QF2 with four biological replicated plants per genotype and WT. Mock-control plants treated with10 mM MgCl_2_ solution. **(B)** Images shown represent symptoms observed six to seven days after treatment with bacterial pathogens CMN, SA and PM.


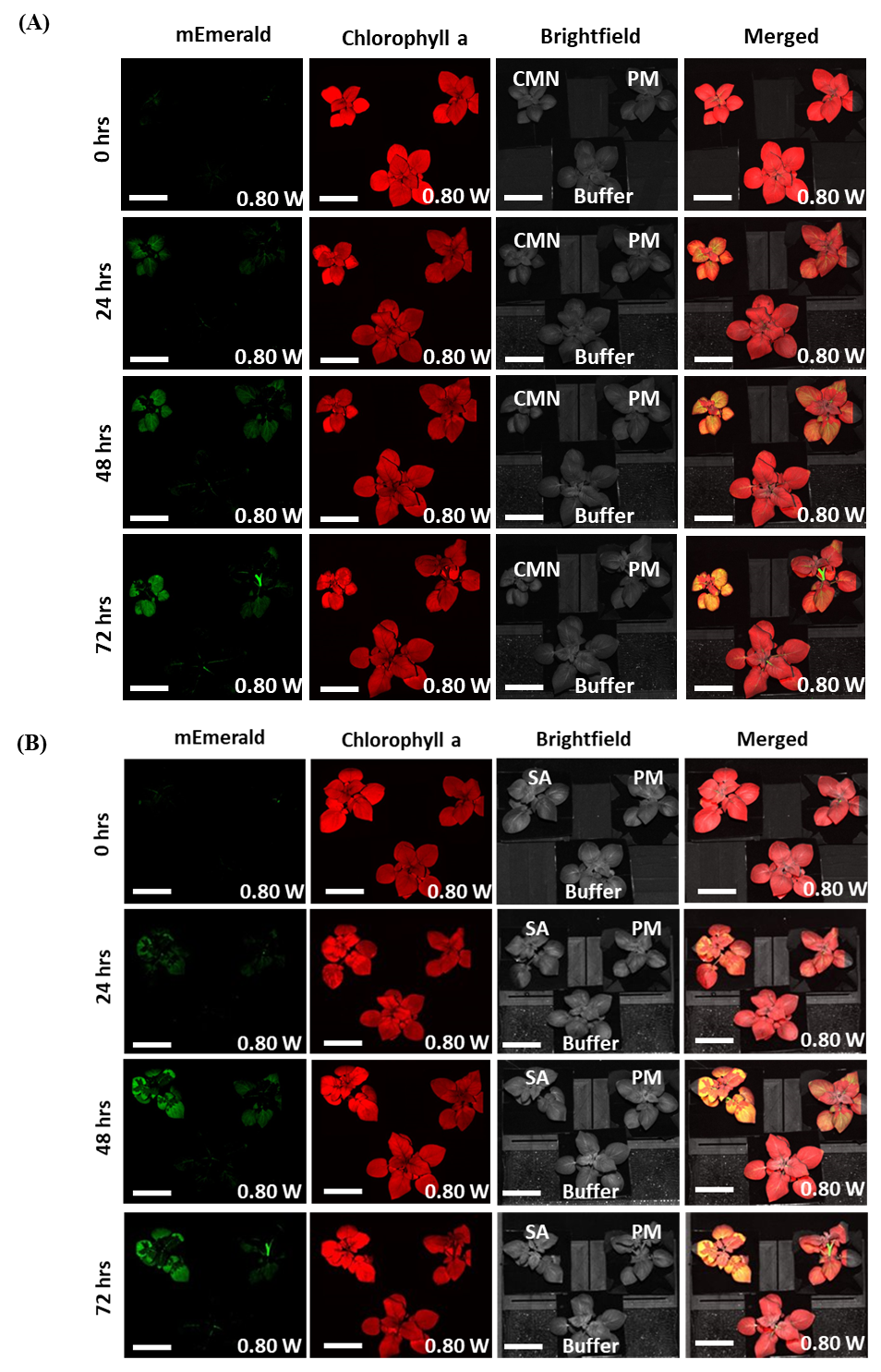


**Figure S3: Time course analysis of mEmerald emission in enhanced pathogen phytosensor.**

Images acquired using the fluorescence-induced laser projector (FILP) system at a distance of 3 m from laser source with 200 ms exposure time at 0.80 watts laser power. mEmerald GFP images were acquired using the 465 nm excitation laser and the 525/50 nm emission filter. Chlorophyll images were acquired using the 465 nm excitation laser and the 680/50 emission filter. One biological replicate for each treatment from a single 4xS-Box:QF2 plant line represented. Three independent experiments were performed with three biological replicates (n=3). Images show the detection of mEmerald at 24, 48, 72 h after inoculation. Pixel intensity at 0 hr was subtracted to normalize against background emission before treatment. Scale bar: 5 cm. **(A)** Transgenic phytosensor 4xS-Box:QF2 (with the Q-system) were inoculated with bacterial pathogens *Clavibacter michiganensis* subsp. *nebraskensis* (CMN), *Pseudomonas marginalis* (PM), and 10 mM MgCl_2_ (buffer mock-control). **(B)** Transgenic phytosensor 4xS-Box:QF2 inoculated with bacterial pathogens *Streptomyces acidiscabie* (SA), *Pseudomonas marginalis* (PM), and 10 mM MgCl_2_ (buffer mock-control). Plants were infiltrated with a bacterial inoculum of OD_600_ = 0.3, corresponding to colony forming units (cfu) of 3 x 10^8^ cfu/ml CMN, 3 x 10^8^ cfu/ml SA, and 1 x 10^8^ cfu/ml PM.

**
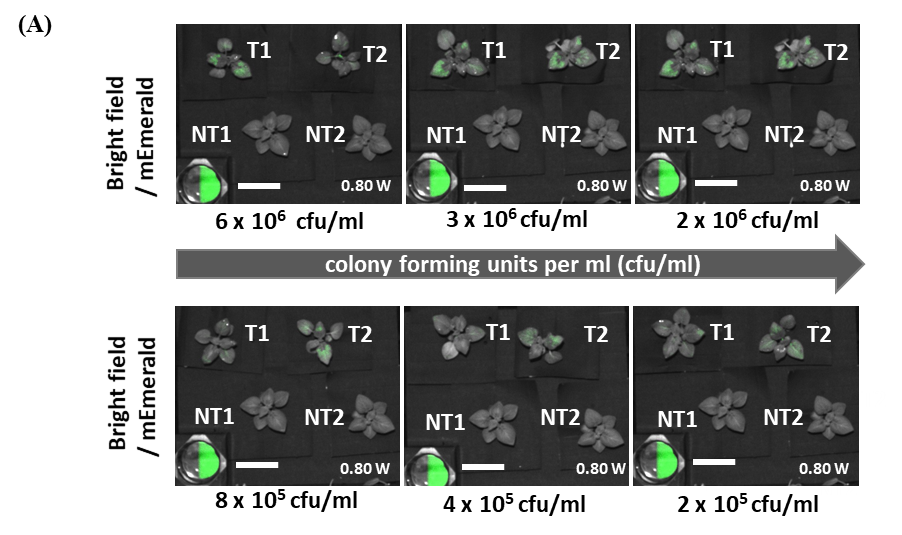
**

**Figure S4: Sensitivity of the enhanced pathogen phytosensor to bacterial pathogen.**

Transgenic phytosensor 4xS-Box:QF2 (with the Q-system) were inoculated with bacterial pathogen *Clavibacter michiganensis* subsp. *nebraskensis* (CMN) at varying cfu/ml. **(A)** Intensity of mEmerald signal at 72 hours post inoculation with indicated cfu/ml of CMN bacteria. Two biological replicates for each treatment from a single 4xS-Box:QF2 plant line represented. Images acquired using fluorescence-induced laser projector (FILP) system, at a distance of 3 m from laser source with 200 ms exposure time at 0.80 watts laser power. mEmerald GFP images were acquired using the 465 nm excitation laser and the 525/50 nm emission filter. Chlorophyll images were acquired using the 465 nm excitation laser and the 680/50 emission filter. NT: non-treated; T: treated. Scale bar: 5 cm.


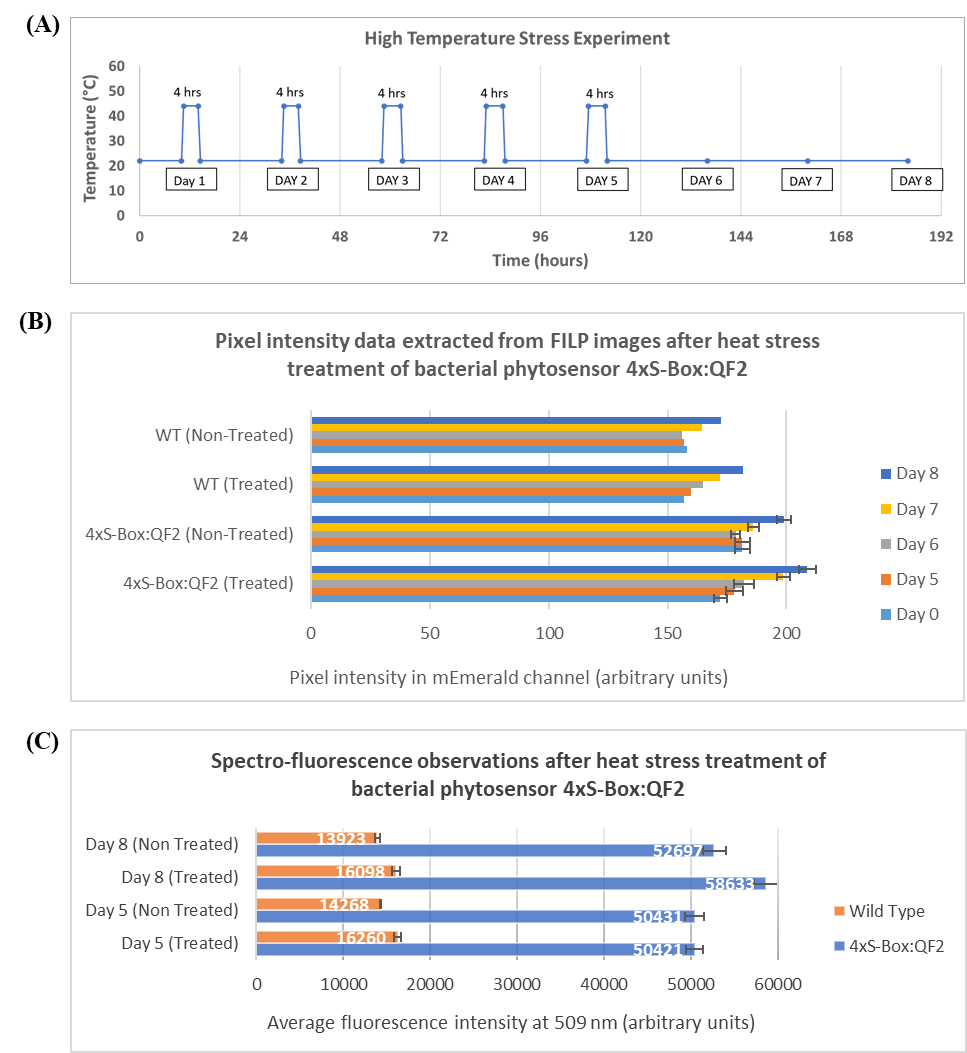


**Figure S5: Response of the enhanced pathogen phytosensor to heat stress treatment.**

Transgenic phytosensor 4xS-Box:QF2 (with the Q-system) and wild type (WT) plants were treated with heat stress. **(A)** Transitory heat stress conditions used for heat treatment. **(B)** Pixel intensity data extracted from images acquired using the fluorescence-induced laser projector (FILP) system, showing mEmerald emission observed after heat stress treatment of 4xS-Box:QF2 compared to 4xSBox and WT (wild type) background served after heat stress treatment. FILP images acquired using the 465 nm excitation laser and the 525/50 nm emission filter at a distance of 3 m from laser source with 200 ms exposure time at 0.80 watts laser power. Day 0 represent FILP images acquired before treatment. Day 5 to Day 8 represent FILP images acquired after heat stress treatment. **(C)** Spectro-fluorescence observations on Day 5 and Day 8, after heat stress treatment. Three technical replicate spectral readings were collected on a single leaf for each biological replicate plant (n = 3) per experiment to account for positional error. Data represent mean ± standard error of 27 readings. No significant difference was observed between treated and non-treated plants (ANOVA, Tukey HSD post hoc).

**Table S1. Sequences of the 4xS-Box promoter fragment used for vector construction.**

| **Name of Sequence** | **Sequence** |
| --- | --- |
| S-Box | CAGCCACCAAAGAGGACCCAGAAT |
| Minimal 35SCaMV promoter | GCAAGACCCTTCCTCTATATAAGGAAGTTCATTTCATTTGGAGAGGA |
| EcoRI-MfeI-4xSBox-minimal-35SCaMV-TMVΩ-AvrII-NcoI | GAATTCAATTGGGTCTCAGGAGTCTAGACAGCCACCAAAGAGGACCCAGAATACTAGACAGCCACCAAAGAGGACCCAGAATACTAGACAGCCACCAAAGAGGACCCAGAATACTAGACAGCCACCAAAGAGGACCCAGAATACTAGTCGCAAGACCCTTCCTCTATATAAGGAAGTTCATTTCATTTGGAGAGGACTATTTTTACAACAATTACCAACAACAACAAACAACAAACAACATTACAATTACTATTTACAATTACCCTAGGCCATGG |

**Table S2. Sequences of primers used for cloning, genotyping plants and qRT-PCR.**

| **Use** |  | **Primer Sequence (5'-3')**​ | **Fragment/Gene amplified** |
| --- | --- | --- | --- |
| Cloning | F | taagcaGAATTCCAATTGGGTCTCAGGAGTCTAGACAGCC | EcoRI-Mfe1-4xSBox:minimal35SCamV:TMVΩ-AvrII-NcoI |
|  | R | cgctgtCCATGGCCTAGGGTAATTGTAAATAGTAATTGTAATGTTGTTTGTTGTTTGTTG |  |
| Genotyping transgenic plants | F | CACCATGGTGAGCAAGG | mEmerald |
|  | R | GATCTAGTAACATAGATGACACCG |  |
|  | F | taagcaGAATTCCAATTGGGTCTCAGGAGTCTAGACAGCC | S-Box-Emerald |
|  | R | TCTCGTTGGGGTCTTTGCTC |  |
|  | F | taagcaGAATTCCAATTGGGTCTCAGGAGTCTAGACAGCC | S-Box-QF2 Transcription factor |
|  | R | GGATCGACTAGTTTAGAGGAGGCGGGTAATGCTC |  |
| qRT-PCR | F | GACCACTACCAGCAGAACAC | mEmerald |
|  | R | TCTCGTTGGGGTCTTTGCTC |  |
|  | F | ATTGGAAACGGATATGCTCCA | Elongation factor 1-alpha |
|  | R | TCCTTACCTGAACGCCTGTCA |  |

Forward primer (F) and reverse primer (R).
